# High-Pressure Homogenized Seaweed Cellulose Nanofibrils-Based Emulsion Gel: An Innovative Platform for Fucoxanthin Encapsulation and Stability Improvement

**DOI:** 10.3390/foods14193338

**Published:** 2025-09-26

**Authors:** Mingrui Wang, Ying Tuo, Yixiao Li, Qianhui Xiao, Yue Liu, Long Wu, Hui Zhou, Yidi Cai, Yuqing Zhang, Xiang Li

**Affiliations:** 1 College of Food Science and Engineering, Dalian Ocean University, Dalian 116023, China; 19741157174@163.com (M.W.); ty15842784797@163.com (Y.T.);; 2 Dalian Jinshiwan Laboratory, Dalian 116034, China; 3 National R&D Branch Center for Seaweed Processing, Dalian Ocean University, Dalian 116023, China; 4 State Key Laboratory of Marine Food Processing & Safety Control, Qingdao 266000, China

**Keywords:** brown seaweed, nanocellulose, fucoxanthin, emulsion, stability

## Abstract

Poor solubility and bioavailability have limited the application of fucoxanthin and functional food processing. In order to encapsulate fucoxanthin in delivery systems, cellulose nanofibril-stabilized emulsion gels (CNFs) derived from industrial brown seaweed residue were developed to enhance fucoxanthin delivery. Cellulose nanofibrils (CNFs) were isolated using high-pressure homogenization at 105 MPa through 5, 10, and 15 cycles (denoted as C5, C10, and C15) and yielding reduced crystallinity down to 52.91 ± 2.13% (C15). The minimum particle size of the present CNFs is approximately 37 nm (C15). Moreover, single-factor and orthogonal experiments optimized the stability of the present emulsion. A 17.5 mg/mL CNFs 50% oil phase with coconut oil, 0.5 mg/mL fucoxanthin, and homogenization for 60 s were identified to be the optimal conditions for such emulsion gel. The present emulsions demonstrated a high storage stability at 4 °C versus 25 °C, which maintained minimal phase separation over 8 days. The release kinetics showed significant dependencies with fucoxanthin release increasing to 9.22 ± 0.62% at pH 8.0, 9.52 ± 0.58% under 1000 mM NaCl, and 8.25 ± 0.62% at 100 °C. In addition, the CNFs effectively preserved the antioxidant activity of the fucoxanthin under different pH values, salinities, and temperatures. The results establish seaweed-derived CNFs as effective stabilizers for fucoxanthin encapsulation, enhancing stability while preserving functionality against food-processing stresses. To our knowledge, no prior research has been reported on a fucoxanthin delivery system utilizing an emulsion gel stabilized by cellulose nanofibrils (CNFs). Such emulsions might provide a sustainable strategy for valorizing seaweed waste and advance functional food applications of marine bioactives.

## 1. Introduction

Fucoxanthin is found abundantly in brown seaweed, and it is a kind of biologically effective carotenoid, that is why it has received much attention for its various health benefits [1,2]. Fucoxanthin is an extremely powerful antioxidant, which can neutralize free radicals and mitigate diseases related to oxidative stress [3]. Also, fucoxanthin can reduce lipid synthesis and increase the consumption of lipids [4]. Fucoxanthin possesses anti-inflammatory properties that can help alleviate chronic inflammation [5], while the anti-tumor activities of fucoxanthin have potential applications in cancer prevention and treatment [6]. But fucoxanthin has many practical uses as an additive in functional foods, medicines, etc., due to its poor solubility and absorption [7]. Additionally, fucoxanthin is susceptible to degradation under heat, oxygen, alkaline pH, and light conditions during processing and storage [8].

To address these challenges, nano-encapsulation strategies have emerged as a promising solution. Among various delivery systems, emulsion gels stabilized by edible solid particles are becoming more and more interesting [9,10,11]. Compared to conventional surfactant-based emulsions, emulsion gels demonstrate remarkable stability, effectively resisting both coalescence and Ostwald ripening, leaving minimal surfactant residue, and having programmable release characteristics [12,13]. CNFs made from seaweed residues are used as stabilizers for emulsion gels. Such CNFs have a high aspect ratio, are bio-degradable, and have tunable surface chemistries, thus they are effective. The biodegradability and tunable surface chemistry of these nanofibrils contribute to their effectiveness [14]. The properties of CNFs enable them to form stable interfacial films, thus making them bioavailable and stable, and, furthermore, making the active compounds included in them bioavailable [15].

Brown seaweed generates a substantial amount of industrial waste after alginate extraction, with cellulose content exceeding 90% [16]. However, the potential of utilizing this waste for CNFs production and fucoxanthin delivery remains largely unexplored. High-pressure homogenization is a scalable and environmentally friendly method. The technique can defibrillate cellulose into CNFs with controlled crystallinity, degree of polymerization, and surface charge [17,18]. The process not only reduces the particle size of the cellulose but also enhances its surface area and reactivity, making it more effective as a stabilizer in emulsion gels [19].

This study isolated the CNFs from industrial brown seaweed waste using high-pressure homogenization. The study proved that there is a change in the different homogenization cycles in the physicochemical properties of CNFs. Furthermore, the study developed an oil-in-water fucoxanthin-loaded emulsion gel that is optimized for encapsulation efficiency, storage stability, and antioxidant activity under typical food-processing conditions. A highly effective novel strategy for fucoxanthin delivery involves its encapsulation within cellulose nanofibrils (CNFs) derived from brown seaweed, offering promising applications in pharmaceuticals, functional foods, and related fields. This innovative system demonstrates superior performance compared to conventional delivery vehicles by ensuring greater stability and robustness, thereby facilitating the effective incorporation of functional ingredients into food products and enhancing the bioavailability and efficacy of bioactive compounds. Also, it promotes sustainability, delivering carriers of bioactive elements for functional food and fills among the lab novelties and practical operations. As far as we all know, this is the first study on emulsions exclusively stabilized by cellulose nanofibrils derived from industrial brown seaweed waste, an underutilized resource with over 90% cellulose content. Unlike conventional systems using non-waste-derived stabilizers or surfactants, our formulation employs high-pressure homogenization to tailor CNFs’ crystallinity, polymerization degree, and surface charge, optimizing interfacial coating for superior resistance to coalescence and Ostwald ripening. The waste-sourced CNFs synergistically provide programmable release and exceptional storage stability under food-processing conditions, thereby addressing critical limitations of poor solubility, environmental degradation, and low absorption inherent in prior delivery systems.

## 2. Materials and Methods

### 2.1. Materials

The cellulose used in this study was sourced from the residual biomass generated after alginate isolation, which was kindly supplied by Qingdao Mingyue Group (Qingdao, China). Brown algal biomass waste from industrial production included glucan content 94.52 ± 7.90%, Ash content 2.63 ± 0.53%, and Moisture content 90.06 ± 0.07%. Fucoxanthin (analytical grade) was sourced from Qingdao Mingyue (Qingdao, China), and coconut oil (food grade) was obtained from Wenchang Dongjiao Coconut Processing Professional Cooperative (Wenchang, China).

The chemicals of anhydrous ethanol, glacial acetic acid, tert-butanol, KPS, Cu-EDTA sol’n, CaCO_3_ KBa(NClO), NaCl, Glucose, and NaOH were of analytical grade and provided by MackLin Co., Ltd. (Shanghai, China). The 2,2-diphenyl-1-picrylhydrazyl (DPPH) used in this study was provided by Hefei Gansheng Technology Co., Ltd. (Hefei, China). Disodium hydrogen phosphate was provided by Wuxi Yatai United Chemical Co., Ltd. (Wuxi, China). Potassium ferricyanide was provided by Tianjin Huasheng Chemical Reagent Co., Ltd. (Tianjing, China), and trichloroacetic acid was provided by Shanghai McLynn Biochemical Co., Ltd. (Shanghai, China).

### 2.2. Brown Seaweed Cellulose Extraction

After processing the raw material (seaweed residue) with a colloid mill, water was removed by filtration through a filter cloth. The residue was then placed in a glass reactor and stirred with an equivalent amount of anhydrous ethanol for 12 h, followed by filtration to remove the ethanol. Then add 20 mL of 0.5 M NaOH solution and react at 75 °C for 6 h. This was performed after being treated with alkali and water washing. Wash with deionized water until neutral. The bleaching step involves adding its volume of deionized water, adjusting the pH to 2.5 with acetic acid, adding sodium chlorite in 1:0.35 (dry mass) ratio, and stirring at 75 °C for 4 h. Wash with deionized water until neutral; filter off. We went through an alkaline process again as well as yet another bleaching step. Finally, we added 1.5 mol/L hydrochloric acid at a ratio of 1:20, stirring and heating it at 100 °C for 20 min. Heating was stopped, and stirring continued at room temperature for 12 h. After washing to neutrality, the final brown seaweed cellulose (BC) sample was obtained.

### 2.3. Preparation of CNFs

High-pressure homogenization was employed to produce cellulose nanofibrils (CNFs) from brown seaweed-derived cellulose. A 15 mg/mL aqueous suspension of the cellulose was prepared in deionized water and acidified to pH 2.5 prior to processing. The suspensions were then processed using a high-pressure homogenizer (AH-BASIC, GEA Niro Soavi, Parma, Italy) operating at 105 MPa, with 5, 10, or 15 passes (designated as C5, C10, and C15, respectively) to produce CNFs with different fibrillation levels. The resulting CNF suspensions were stored at 4 °C until further use.

### 2.4. Determination of Particle Size of CNFs

Different homogenization cycle samples of BC and CNFs (C5, C10, and C15) were analyzed by a nanoparticle size analyzer (Nano-ZS90, Malvern, Jessup, UK). Take 1.0 mL with an amount of 13.8 mg/mL of the sample and put it into the cuvette for measurement. CNF samples were measured with incident angle 90° and refraction index 1.330 at 25 °C. The particle size and polydispersity index (PDI) of CNFs were then measured using the Zeta Sizer Software (Malvern Panalytical 3.3.0. Malvern, Jessup, UK).

### 2.5. Fourier Transform Infrared Spectroscopy (FTIR) Analysis of CNFs

Fourier Transformation Infrared Spectroscopy (FTIR) was selected to detect the chemical structure of the different sample solutions BC, C5, C10, and C15. A 3.0 µL portion of the powder from the dried sample solution was evenly added to 0.2 g of potassium bromide and then ground into a fine powder. Then the samples were pressed into tablet samples and placed in the instrument sample compartment. It was then analyzed by a FTIR Spectrometer (NICOLET iS50, Thermon Scientific, Waltham, MA, USA). FTIR spectra were acquired in transmission mode from 4000 to 400 cm^−1^ at a resolution of 4 cm^−1^, accumulating 32 scans per spectrum

### 2.6. Crystal Structure Analysis

The crystal structure and crystallinity index of the CNFs samples were analyzed using X-ray diffraction (XRD). The samples were analyzed at 40 kV and 40 mA, with a scattering angle range of 5–60°. The crystallinity index (CrI) was calculated using the following formula:

(1) $$CrI=\frac{\left(I_{c}-I_{a}\right)}{I_{c}}\times 1$$

where *I_c_* is the diffraction intensity at 2θ = 22.5° (crystalline region), and *I_a_* is the diffraction intensity at 2θ = 16.5° (amorphous region).

### 2.7. Effect of the CNFs Concentration on the Stability of Emulsion Gels

The CNF dispersions at concentrations of 5, 10, 15, and 20 mg/mL were CNF dispersions with coconut oil as 4:1 (volume ratio). Mixtures with a speed of 7500 rpm were homogenized for 45 s in order to make the emulsion. The samples were kept at 4 °C for 4 h and then spun once more for 30 s at 4000 rpm (4 °C). The creaming index is calculated using the following formula:

(2) $$CI=\frac{U}{T}\times 100\%$$

where *CI* is the creaming index (%), *U* is the height of the upper layer (cm), and *T* is the total height of the emulsion (cm).

### 2.8. Effect of the Homogenized Time on the Stability of the Emulsion Gel

CNF dispersions (c = 15 mg/mL) and coconut oil were mixed with a 4:1 volume ratio. The mixtures were homogenized at 7500 rpm for 45, 60, and 75 s to form emulsions. The emulsions were stored and centrifuged as described in Section 2.7. The creaming index was calculated using Formula (2).

### 2.9. Effect of the Oil-Phase Volume Fraction on the Stability of the Emulsion Gel

CNF dispersions (15 mg/mL) were combined with fucoxanthin-loaded coconut oil to form emulsions with oil-phase volume fractions of 15%, 20%, 25%, and 30%. The mixtures were homogenized at 7500 rpm for 45 s. The emulsions were stored and centrifuged as described in Section 2.7. The creaming index was calculated using Formula (2).

### 2.10. Orthogonal Experiment

According to the experimental results of the single-factor experiments, the optimal conditions for brown seaweed CNFs’ mass fraction, homogenization time, and oil-in-water-phase volume fraction were selected. A three-factor, four-level orthogonal experimental design (as shown in Table 1) was subsequently conducted to systematically optimize the emulsion preparation parameters.

### 2.11. Preparation of CNFs-Stabilized Fucoxanthin Emulsion

Fucoxanthin-enriched coconut oil was prepared by dissolving fucoxanthin in the oil at concentrations of 0.125, 0.250, and 0.500 mg/mL. Based on the optimal conditions determined by orthogonal experiments, preferred fucoxanthin emulsions were prepared. A 20 mL brown seaweed CNFs suspension (15 mg/mL) was homogenized with the fucoxanthin-enriched coconut oil using a homogenizer (GEA Niro Soavi, Parma, Italy) at 7500 rpm for 60 s to obtain brown seaweed CNF-stabilized fucoxanthin emulsions. Three parallel emulsion samples were prepared for each concentration. Finally, a 4 h incubation at 4 °C was used to stabilize the fucoxanthin emulsion samples, followed by image capture using a digital camera (COOLPIX P900s, Nikon, Tokyo, Japan).

### 2.12. Storage Stability of Emulsions

The emulsion gels containing different fucoxanthin concentrations were stored at 4 °C and 25 °C for periods of 8, 18, and 28 days. Prior to determining the creaming index, the samples were equilibrated at room temperature (25 °C) for 1 h, after which the creaming index was measured following the procedure outlined in Section 2.7.

### 2.13. Determining the Interfacial Adsorption Capacity of CNFs

The quantification of CNFs adsorbed at the interface was conducted using a centrifugation-based method adapted from an established protocol [20]. Briefly, 5 g of the fucoxanthin emulsion was centrifuged at 5000 r/min for 6 min. The CNFs were subsequently isolated from the supernatant and dried until a constant mass was attained, herein referred to as C_S_. The interfacial adsorption was subsequently calculated according to the following formula:

(3) $$Adsorption=100-\frac{C_{s}}{C_{0}}\times 100\%$$

where C_S_ denotes the concentration of CNFs remaining in the supernatant after centrifugation of the Pickering emulsion, and C_0_ corresponds to the initial concentration of CNFs present in the aqueous phase.

### 2.14. Determination of Fucoxanthin Release Rate in the Emulsion Gel

The emulsion gel was extracted with petroleum ether after 7 days of storage at room temperature (25 °C) to obtain fucoxanthin, and the concentration of fucoxanthin was measured using a spectrometer at 450 nm. The release rate was calculated using the following formula:

(4) $$X=1-\frac{\left(A\right)}{C}\times 100\%$$

*X* is the release rate (%), *A* is the measured concentration of fucoxanthin (mg/mL), and *C* is the initial concentration of fucoxanthin in the emulsion (mg/mL).

### 2.15. Formulating Fucoxanthin Emulsions at Different pH Levels

This study evaluated the robustness of the fucoxanthin emulsion gel by subjecting it to a series of environmental stresses relevant to food processing, such as diverse pH levels, salinity, thermal treatments, and UV exposure. For the preparation of the emulsions in different pH values, 24 g of cellulose was placed in 4 separate 20 mL beakers, adjusting the solution pH with 0.5 mol/L sodium hydroxide and hydrochloric acid to pH 3.0, 5.0, 7.0, 9.0, and 11.0, respectively. Afterwards, a fucoxanthin emulsion gel was achieved under good conditions, as mentioned in Section 2.11. After the exposure emulsion was stored at room temperature for 7 days, fucoxanthin emulsions with different pHs were made in accordance with the process given in Section 2.14.

### 2.16. Influence of Salinity on the Liberation Kinetics of Fucoxanthin

To prepare fucoxanthin emulsions with varying salinity, 200, 400, and 600 mmol/L of sodium chloride were added to CNF suspensions, respectively (pH = 7.0), and samples were stored in uncovered containers at room temperature for 7 days. Free fucoxanthin was extracted by the method described in Section 2.14, and the release amount of fucoxanthin was obtained.

### 2.17. Effect of Temperature on the Release Rate of Fucoxanthin

Place the 1 mL fucoxanthin emulsion gel in 1.5 mL centrifuge tubes. Then, heat the fucoxanthin emulsion gel in water baths at 50 °C, 75 °C, and 100 °C for 10 min. After heating, quickly place them in a centrifuge and centrifuge at 8000 rpm and 4 °C for 5 min. Immediately obtain the free fucoxanthin by the method described in Section 2.14, and calculate the release rate of fucoxanthin.

### 2.18. DPPH Free Radical Scavenging Activity of Fucoxanthin Within the Emulsion Gel System

As outlined in Section 2.14, Section 2.15, Section 2.16 and Section 2.17, the DPPH radical scavenging activity of purified fucoxanthin was assessed using an established protocol [21]. Briefly, 0.3 g of the fucoxanthin-loaded emulsion gel was mixed with 1.2 mL of ethanolic DPPH solution and incubated in the dark at 25 °C for 30 min. After incubation, the mixture was centrifuged at 10,000 rpm for 10 min. The absorbance of the supernatant (*A_i_*) was measured at 517 nm using an Epoch 2 microplate reader (BioTek, Winooski, VT, USA). Control samples were prepared by mixing 0.3 g of the fucoxanthin emulsion gel with 1.2 mL of 95% ethanol (*A_j_*) and 0.3 g of 95% ethanol with 1.2 mL of the DPPH solution (*A*_0_). The DPPH radical scavenging rate was calculated with Equation (5):

(5) $$DPPH\;free\;radical\;scavenging\;rate\;\left(\%\right)=\left(1-\frac{A_{i}-A_{j}}{A_{0}}\right)\times 100\%$$

### 2.19. Ferric Ion Reducing Capacity (FIRC) of the Fucoxanthin in Emulsion Gel

The FIRC analysis of the isolated fucoxanthin (Section 2.14, Section 2.15, Section 2.16 and Section 2.17) was performed in accordance with an established protocol. Disodium hydrogen phosphate was prepared [22]. Sodium dihydrogen phosphate was used to prepare a 0.2 MPBS solution with pH 6.6. Furthermore, the 1% potassium ferricyanide solution, the 10% trichloroacetic acid solution, and the 0.1% ferric chloride solution were also prepared. According to the grouping specified in Section 2.14, Section 2.15, Section 2.16 and Section 2.17, the 0.5 mL of PBS buffer solution (0.2 M, pH 6.6) and 0.2 mL of 1% potassium ferricyanide solution were added to each group. The mixture was then subjected to a reaction at 50 °C in a constant-temperature and humidity-controlled digital water bath (HH-8J, Langyue, Changzhou, China) for 20 min. We added 1 mL of the 10% trichloroacetic acid solution into the mixture and then centrifuged at 3000 r/min for 10 min. Take the supernatant and ferric chloride solution as 0.5 mL of the sample solution, add 0.5 mL of pure water and 0.1 mL of 0.1% ferric chloride solution. Mix them up and let stand for 10 min. Finally, we took the absorbance of the solution at 700 nm. In each sample, the experiment was carried out, and the FRAP was calculated with the use of Formula (6):

(6) $$A=A_{1,2}-A_{0}$$

where A is the amount of reduction, A_1_ and A_2_ are the absorbance readings of the sample and the standard, respectively, and A_0_ is the absorbance value of the blank

### 2.20. Statistical Analysis

Calculate the averages and standard deviations for every treatment: We used ANOVA (Analysis of Variance) through SPSS software package (SPSS16.0, IBM, Armonk, NY, USA) to determine whether there was a significant difference (*p* < 0.05) among the different treatments given. Data plotting was performed using Origin 2018.

## 3. Results and Discussion

### 3.1. Characterization of CNFs

#### 3.1.1. Effect of Homogenization Cycles on Particle Size of Cellulose Nanofibers

**Figure 1:**
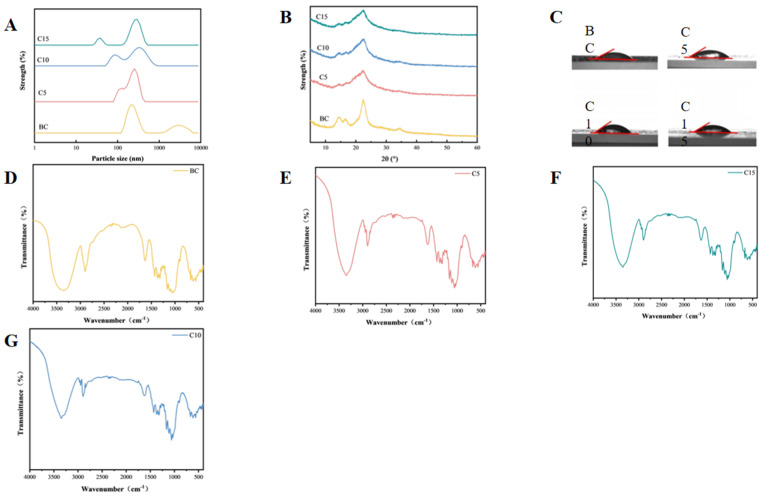
Characteristics of cellulose nanofibrils subjected to varying homogenization cycles. (**A**) Particle size of CNFs; (**B**) X-ray diffraction patterns of CNFs; and (**C**) contact angles of CNFs. (**D**) FTIR spectrums of CNFs in BC sample; (**E**) FTIR spectrums of CNFs in C5 sample; (**F**) FTIR spectrums of CNFs in C10 sample; and (**G**) FTIR spectrums of CNFs in C15 sample.

High-pressure homogenization breaks the hydrogen bonds between cellulose molecules, reduces the size, and promotes fibrillation [23]. According to Figure 1, the results confirm that the result shows that the number of high-pressure homogenization cycles is a large factor affecting the brown seaweed cellulose particle size. The minimum particle size of sample C5 is around 91 nm, confirming that the brown seaweed CNFs sample was obtained by high-pressure homogenization at 105 MPa for five cycles in the experiment. Sample C10 has a diameter of 78 nm, and sample C15 has a diameter of 37 nm. The gradually decreasing diameter with the number of homogenization cycles may be attributed to the shear cavitation from the influence of high-pressure homogenization, which disrupts the hydrogen bonds between cellulose molecules, leading to a reduction in sample particle size through fracture or defibrillation [24]. This result was supported by the relevant research conclusion reported by Tanja et al. [25,26,27], in which high-pressure homogenized wheat straw pulp was homogenized four, six, and seven times at 105 MPa to obtain CNFs, with a particle size distribution within the range of 10 to 100 nm. The result shows that, through the process of high-pressure homogenization, it can greatly reduce the size of the cellulose to become CNFs. The polydispersity index (PDI) values provided in Table 2 further support the effect of homogenization on the uniformity of CNF size distributions. As the number of homogenization cycles increases, the PDI decreases gradually from 0.64 ± 0.01 for the untreated sample (BC) to 0.57 ± 0.02 for the sample subjected to 15 cycles (C15). This reduction in the PDI indicates a narrower and more uniform size distribution of the CNFs, reflecting an improved fibrillation and defragmentation efficiency with repeated homogenization passes [28]. The trend aligns with the observed decrease in particle size, suggesting that higher numbers of homogenization cycles not only reduce fiber dimensions but also enhance the homogeneity of the resulting nanofibers.

#### 3.1.2. Effect of Homogenization Times on the Chemical Structure of the CNFs

Figure 1B is the FTIR of CNFs. Homogenized CNFs show the same FTIR as the raw CNFs but with slight changes in some bands with the peak of 3350–3175 cm^−1^ in the sample FTIR spectrum representing the -OH and the peak at 2898 cm^−1^ representing -CH_2_-. The peak at 1628–1623 cm^−1^ shows the -OH absorption, at 1420–1460 cm^−1^ a -CH functional group, and at 1059 cm^−1^ a C-O from the sample is shown. The characteristic functional groups of cellulose were observed in all samples. Compared with the BC samples, no changes in functional group structure were found in the CNF samples. Meanwhile, with the increase in the homogenization times, no changes in the functional group structure were observed. Such results were supported by the research results of Chen Wenshuai et al. [29]. They prepared nanocellulose from poplar wood powder and conducted relevant chemical structure analysis on it. The result proves that what you extract is cellulose, which is a good starting material for us to further explore the study of emulsion gels. The absence of characteristic peaks in the 400–800 nm range for both BC and CNFs indicates a lack of detectable fucoxanthin, which is likely attributable to its degradation during industrial alginate extraction. This degradation implies that any potential surface-active or antioxidant properties contributed by fucoxanthin would be absent in these samples. Consequently, the stabilization of the emulsion gel must be wholly attributed to the polysaccharide components (cellulose and alginate-residual fractions) rather than any surfactant-like effect from carotenoids. The loss of fucoxanthin could therefore simplify the interpretation of stabilization mechanisms, confirming that it is driven purely by CNF-based interfacial adsorption and network formations.

#### 3.1.3. Effect of Homogenization Cycles on Crystalline Structure of Brown Seaweed Cellulose

The XRD patterns of the brown seaweed CNFs are presented in Figure 1C. The diffraction peaks of all samples appeared at approximately 2θ = 14.5°, 16.5°, and 22.5°, confirming the typical cellulose I crystalline structure. Notably, the CNFs samples (C5, C10, and C15) exhibited sharper diffraction peaks compared to the non-homogenized BC samples.

As summarized in Table 2, the crystallinity index (CrI) of the homogenized CNFs was significantly reduced relative to the untreated sample. This decrease in crystallinity is likely attributable to the disruption of the crystalline regions caused by the high shear and cavitation forces during homogenization. As the number of homogenization cycles was raised from 5 to 15, a progressive reduction in the CrI was observed. Specifically, sample C15 showed the lowest crystallinity of 52.91 ± 2.13, representing a 12.12% decrease compared to the non-homogenized sample BC (60.21 ± 4.32). These findings are consistent with those reported by Fang Lei et al. [30], although the absolute values were approximately 34% higher in the present study.

#### 3.1.4. Contact Angle of Cellulose Nanofibers as a Function of Homogenization Pressure

The interfacial contact angle is defined as the angle formed at the junction of a liquid phase, solid surface, and second immiscible fluid phase [31]. It results from the equilibrium of the three interfacial tensions and quantitatively characterizes the wettability of the solid surface by the liquid [32]. A contact angle below 90° is characteristic of a hydrophilic surface, in contrast to an angle exceeding 90°, which indicates hydrophobicity [33].This kind of angle is crucial in order to understand the attraction between the solid surface and the aqueous phase or the oil phase, as that will be responsible for the stability and kind of emulsion gel such as oil-in-water or water-in-oil [34]. From Figure 1D it can be noted that the number of high-pressure homogenization cycles does not affect the interfacial characteristics of CNF samples.

The measured interfacial contact angle results are shown in Table 2. Compared with the BC samples, the interfacial contact angle of the homogenized group increased, indicating that the hydrophilicity of the CNF samples after homogenization significantly increased. As shown in Table 3, the interfacial contact angle of C10 was the highest (48.0 ± 1.8). The reason might be that the change in molecular structure disrupted the internal cross-linking of cellulose molecules. With the growth in the number of samples being homogenized, the angle formed by the interface of the samples first goes up and then turns down [35].

### 3.2. Optimization of Emulsion Gel Formulation with the CNFs

#### 3.2.1. Effect of the CNF Concentrations on Emulsion Gel Stability

**Figure 2:**
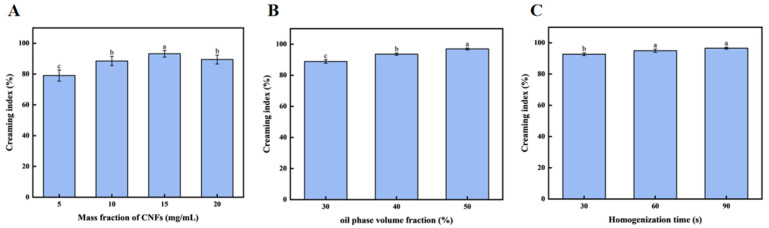
Optimization of emulsion gel formulation with the CNFs. (**A**) The effect of the mass fraction of CNFs on the stability of emulsion gels; (**B**) the effect of oil-phase volume fraction on the stability of emulsion gels; and (**C**) the effect of homogenization time on the stability of emulsion gels. Different letters (a–c) indicate significant differences between groups (*p* < 0.05).

As seen in Figure 2A, the stability of the emulsion gel improved as CNF concentrations increased, until 15 mg/mL, after which it decreased to 20 mg/mL. The highest stability was observed at 15 mg/mL CNFs, indicating that an optimal concentration of CNFs is necessary for maximum emulsion gel stability. It is in line with the result of Hu Zhen et al., in which they found that, as the number of CNFs increased to 0.25 wt%, the stability was significantly improved. However, the rate of coalescence, as indicated by the rate of change in backscattering, was lowest for the 0.5 wt% CNFs emulsion gel. It is indicated that insufficient coverage of CNFs at low concentrations leads to instability, while excessive concentrations of CNFs result in depletion flocculation [35].

#### 3.2.2. Effect of Oil-Phase Volume Fraction on Emulsion Stability

As shown in Figure 2B, the emulsion is stable at a higher phase ratio of oil at 0–50% and then becomes unstable. The highest stability was observed at the 50% oil-phase volume fraction, indicating that an optimal oil-phase volume fraction is necessary for maximum emulsion gel stability. This optimal ratio corresponds to a critical volume fraction of the internal phase, which maximizes the droplet packing density and minimizes coalescence, thereby enhancing stability. This result is similar to that reported by YueYing Pan et al. [36], where in such emulsion gels with an oil-phase volume fraction of 70%, they exhibited the best stability in their water-in-oil emulsion gel system, while those with a lower or higher oil-phase volume fraction showed decreased stability. The observed difference in optimal oil phase volume fraction (50% versus 70%) underscores the fundamental role of the internal-phase volume fraction in determining emulsion gel stability. The nature and amount of the internal phase critically influence stability through increased viscosity, modified droplet interactions, and stabilization of the interfacial film [37].

#### 3.2.3. Effect of Homogenization Time on Emulsion Gel Stability

As shown in Figure 2C, the emulsion gel stability improved with homogenization time, reaching a maximum at 60 s. The stability was the highest at 90 s of homogenization, but there was no significant difference at 60 s. In order to simplify the experiment, 60 s was chosen for further study, indicating that sufficient homogenization time is necessary for maximum emulsion gel stability. This is similar to the content studied by Carsten G et al. [38] and others. They found that increasing the number of homogenization cycles could also reduce particle size, but the effect was relatively small. This indicates that the parameter optimization of the homogenization process is crucial for emulsion gel stability.

#### 3.2.4. Optimal Emulsion Gel Formulation

Based on the results of the single-factor experiments, this study selected three optimization factors: oil-phase volume fraction, the mass fraction of CNFs, and homogenization time. An orthogonal experimental table was then constructed with these three factors at four levels, as shown in Table 4. The orthogonal experiment was conducted using the creaming index of the emulsion gels stabilized with CNFs as the response value. After analyzing the data using SPSS 16.0 software, a one-way ANOVA was used for inter-group comparisons, and the results are shown in Table 4. The optimization results indicate that the mass fraction of CNFs is the most significant factor affecting the stability of the emulsion gel, followed by the oil-phase volume fraction and the homogenization time. The optimal combination for constructing the emulsion gels stabilized with CNFs is the mass fraction of CNFs of 17.5 mg/mL, a homogenization time of 45 s, and an oil-phase volume fraction of 50%. Based on this condition, subsequent experiments were conducted in the study.

### 3.3. Storage Stability of Fucoxanthin Emulsion Gels

**Figure 3:**
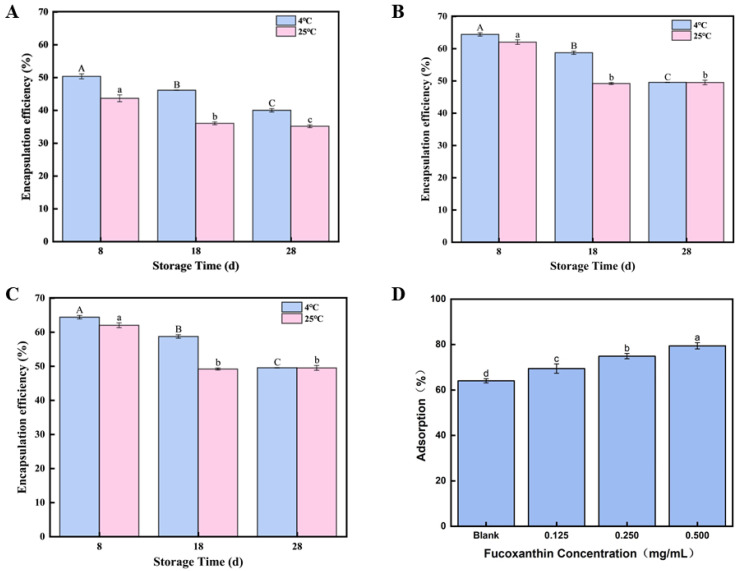
Storage stability of fucoxanthin-loaded emulsion gels: (**A**) storage stability of 0.125 mg/mL fucoxanthin emulsion gel for 8, 18, and 28 days; (**B**) storage stability of 0.250 mg/mL fucoxanthin emulsion gel for 8, 18, and 28 days; (**C**) storage stability of 0.500 mg/mL fucoxanthin emulsion gel for 8, 18, and 28 days; and (**D**) the relative interfacial adsorption. Different letters (A–C, a–d) indicate significant differences between groups (*p* < 0.05).

As shown in Figure 3A–C, different concentrations of fucoxanthin-loaded emulsion gels (0.125, 0.250, and 0.500 mg/mL) were prepared and stored at 4 °C and 25 °C for 8, 18, and 28 days to evaluate their stability. Two control groups containing only oil or fucoxanthin solution were established for comparison, with the left three groups in the image representing control conditions. The experimental results were photographically documented. According to Figure 3, it can be observed that the emulsion gel activity index generally decreased with prolonged storage time, indicating the progressive destabilization of the emulsion gels. This decrease in stability over time aligns with the observed decline in fucoxanthin encapsulation efficiency during storage reported in similar systems [39]. The reduced stability is likely attributed to enhanced Brownian motion among droplets over time, leading to droplet aggregation and size enlargement [40]. Notably, storage at 4 °C resulted in better stability retention compared to 25 °C. This temperature-dependent effect can be partially explained by the solidification of coconut oil within the emulsion gel between 4 °C and 25 °C; this reinforcement of the emulsion gel architecture likely enhances the fucoxanthin encapsulation efficiency at reduced temperatures, thereby possibly delaying its molecular breakdown [41].

A time-dependent decrease in the encapsulation rate was observed in all emulsion gels during 28 days of storage at both 4 °C and 25 °C. The 0.125 fucoxanthin emulsion gel dropped to 43.68% on the 8th day at 25 °C, while the 0.500 mg/mL fucoxanthin emulsion gel maintained optimal encapsulation at 4 °C, reaching 78.73% on the 2nd day and still above 64% on the 8th day. In addition, as shown in Figure 3C,D, after 28 days of storage at 4 °C and 25 °C, emulsion gels of all concentrations exhibited a time-dependent decrease in the creaming index. At 25 °C, the emulsion gel containing 0.125 mg/mL of fucoxanthin showed a creaming index of 40% by day 28, while the 500 mg/mL emulsion gel maintained the best emulsification performance, with a creaming index of 49.53% at 4 °C and also demonstrated a higher creaming index of 40% at 25 °C, compared to other concentrations. The Brownian motion between droplets during storage might be attributed to this result, as it causes the droplets to increase in size and aggregate after collision [42], because Brownian motion causes droplets to bump into each other, making their size bigger, and they clump together. Given that the coconut oil within the emulsion gel can undergo solidification within the temperature range of 14–25 °C [43], this solidification could improve the stability of the emulsion gel, and it could enhance the stability of the emulsion gel. Also, compared to 25 °C, maybe the fucoxanthin of the emulsion gel solidified at 4 °C has a higher proportion. Therefore, the results show that the best strategy is an optimal level of 0.500 mg/mL fucoxanthin, which is stored at 4 °C, and the stability is even better and has a fairly stable system for functional food.

### 3.4. Interfacial Adsorption Capacity of Cellulose Nanofibrils Within the Fucoxanthin-Loaded Emulsion Gel System

The formation of a densely packed interfacial layer by emulsifiers provides substantial steric hindrance and confers long-term stability to the emulsion gel system. Figure 3D illustrates the influence of the fucoxanthin concentration on the relative extent of nanocellulose adsorption at the oil–water interface within the emulsion gel system. Compared to the blank control, the inclusion of fucoxanthin at 0.5 and 1.0 mg/mL significantly enhanced the interfacial adsorption of nanocellulose (*p* < 0.05). However, a further increase in the fucoxanthin concentration to 1.5 mg/mL resulted in a noticeable reduction in nanocellulose adsorption. This trend is consistent with the measured fucoxanthin encapsulation yield achieved in the emulsion gel system. An analogous phenomenon has been reported in curcumin-encapsulated emulsion gels, which demonstrate an enhanced gel rigidity and superior stability relative to systems lacking curcumin. These findings suggest that fucoxanthin within the range of 0.5 to 1.5 mg/mL facilitates the formation of well-developed interfacial CNF films around the emulsion droplets. Owing to the inherent properties of high-pressure homogenized CNFs, their pronounced interfacial adsorption is likely to contribute to the elevated thermal degradation resistance and improved structural integrity of the emulsion gel. Based on these results, the emulsion containing 1.0 mg/mL fucoxanthin was chosen for further analysis.

### 3.5. Effect of Environmental Factors on Fucoxanthin Release Rate

#### 3.5.1. Effect of pH Value on Release Rate of the Fucoxanthin

**Figure 4:**
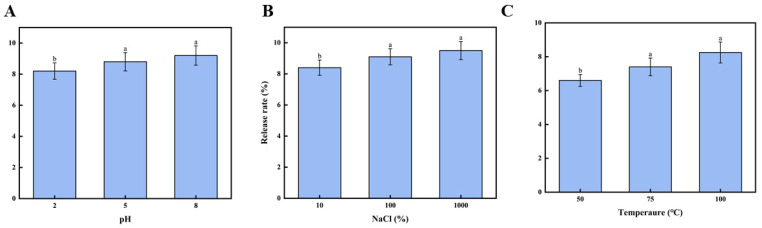
Effect of environmental factors on fucoxanthin release rate: (**A**) effect of pH on fucoxanthin release rate; (**B**) effect of salinity on fucoxanthin release rate; and (**C**) the effect of temperature on the release rate of fucoxanthin. Different letters (a,b) indicate significant differences between groups (*p* < 0.05).

To achieve homeostatic delivery of fucoxanthin in the metabolic system through functional food matrices, the study investigated the encapsulation properties of the brown seaweed CNF emulsion gel systems for fucoxanthin stabilization. The release kinetics of fucoxanthin from emulsion gels under various environmental stimuli were systematically examined to establish a theoretical foundation for targeted delivery systems. As shown in Figure 4A, the pH-dependent release profile of fucoxanthin was evaluated across a physiological pH of 2.0–8.0. The release rate exhibited a progressive increase with the rising pH value, reaching 9.22 ± 0.62% at pH 8.0. This pH-responsive release behavior correlates with emulsion gel stability alterations. The results demonstrate that pH variations significantly modify the surface charge characteristics of emulsion gel droplets, consequently affecting their colloidal stability. The results conclusively establish the pronounced influence of pH on fucoxanthin release from the emulsion gel stabilized by the CNFs.

#### 3.5.2. Effect of Salinity on Release Rate of the Fucoxanthin

Figure 4B demonstrates the release profile of fucoxanthin from emulsion gels under varying sodium chloride or NaCl concentrations, showing a concentration-dependent increase in the release rate from 10 mM to 1000 mM NaCl that reached a maximum of 9.52 ± 0.58% at 1000 mM; these results corroborate the figure findings, substantiating that stability variations originate from modifications in the electrical properties of emulsion gel droplets and conclusively demonstrate the significant impact of salinity on fucoxanthin release kinetics from the emulsion gel stabilized by the CNFs.

#### 3.5.3. Effect of Temperature on Release Rate of the Fucoxanthin

The release rate of fucoxanthin from the emulsion gel under temperatures from 50 °C to 100 °C is shown in Figure 4C. As the heating temperature increased, the fucoxanthin release rate progressively increased [44]. The highest release rate (8.25 ± 0.62%) was observed at 100 °C. Such results might be attributed to emulsion gel stability, suggesting that elevated temperatures compromise the stability of the fucoxanthin emulsion gels. The findings indicate that the temperature exerts a significant (*p* < 0.05) influence on the stability of cellulose nanofibril-stabilized emulsion gels derived from brown seaweed. These results offer a theoretical foundation for utilizing fucoxanthin-loaded emulsion gels in thermally processed foods.

### 3.6. Evaluating the Antioxidant Activity of Fucoxanthin Within the Emulsion Gel System

#### 3.6.1. The DPPH Free Radical Scavenging Activity of the Fucoxanthin in Emulsion Gels

**Figure 5:**
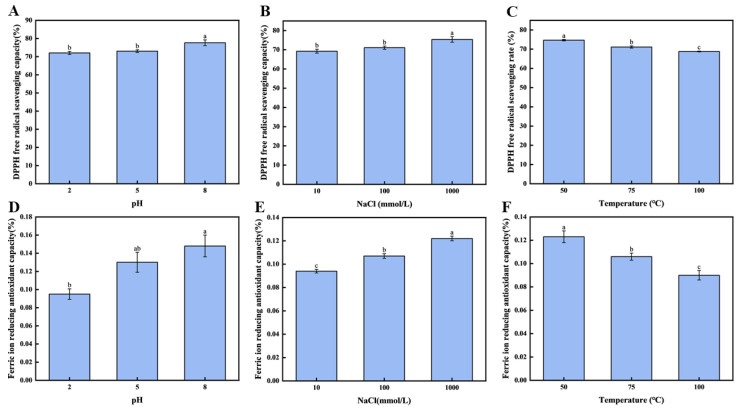
Antioxidant activity of fucoxanthin in emulsion gels: (**A**) the free radical scavenging rate of DPPH under different pH conditions; (**B**) the scavenging rate of DPPH free radicals under different concentrations of NaCl; (**C**) the free radical scavenging rate of DPPH under different temperature conditions; (**D**) the FIRC values under different pH conditions; (**E**) the FIRC values of NaCl at different concentrations; and (**F**) the FIRC values under different temperature conditions. Different letters (a–c, ab) indicate significant differences between groups (*p* < 0.05).

To further evaluate the protective capacity of the brown seaweed CNF emulsion gel-stabilized systems derived from brown seaweed on the antioxidant performance under various conditions, the DPPH radical scavenging rate and the FIRC were assessed across different pH levels and salinities. As illustrated in Figure 5A, when the emulsion gel pH was adjusted from 2.0 to 8.0, a significant enhancement in the DPPH radical scavenging activity of fucoxanthin was observed (*p* < 0.05). The highest scavenging rate, 77.64 ± 1.60%, was achieved at pH 8.0, indicating that the pH considerably influences the antioxidant behavior of fucoxanthin in the emulsion gel.

Similarly, as shown in Figure 5B, the concentration of NaCl exerted a notable effect on the DPPH scavenging capacity (*p* < 0.05). Increasing the NaCl concentration from 10 mM to 1000 mM led to a substantial rise in the scavenging activity, reaching a maximum of 75.37 ± 1.48% at 1000 mM. These findings confirm that ionic strength is a critical factor modulating the antioxidant efficacy of fucoxanthin within the emulsion gel system. The consistency between these results and those pertaining to fucoxanthin release under varying salinity levels suggests that the emulsion gel stability may underlie the observed trends in antioxidant performance. Furthermore, as depicted in Figure 5C, a negative correlation was identified between temperature and the preserved antioxidant activity of fucoxanthin within the emulsion gel (*p* < 0.05). Elevating the temperature from 50 °C to 100 °C resulted in a pronounced reduction in the DPPH radical scavenging rate. The value recorded at 50 °C (74.68 ± 0.40%) was significantly higher than those at elevated temperatures (*p* < 0.05) and also exceeded the 53.53% scavenging rate reported for lutein after 1 h at 40 °C by Bhat et al. [45].

#### 3.6.2. The FIRC of the Fucoxanthin in Emulsion Gels

As the pH and salinity levels increased, the max of the FIRC value (fucoxanthin emulsion gel) was reached at pH 8.0 and a sodium ion concentration of 1000 mM. The pH and salinity greatly impacted the antioxidant ability of fucoxanthin emulsion gels. From Figure 5C, it is clear that the pH greatly affects the FIRC of phycocyanin in the emulsion gel. When the pH value of the emulsion gel increases from 2.0 to 8.0, the FIRC of phycocyanin in the emulsion gel is significantly enhanced (*p* < 0.05). Moreover, the reducing ability rate reaches 0.15 ± 0.02%, which confirms that the pH value can significantly affect the FIRC of phycocyanin in the emulsion gel.

In addition, as shown in Figure 5D, salinity also has a significant effect on the FIRC values of fucoxanthin in the emulsion gel (*p* < 0.05). When the concentration of sodium chloride increases from 10 mM to 1000 mM, the FIRC is significantly improved (*p* < 0.05), which provided the highest level at 1000 mM. Under the condition of a concentration of sodium chloride of 1000 mM, the emulsion gel has the optimal reducing ability, with the FIRC reaching 0.12 ± 0.02%.

As illustrated in Figure 5F, for the effect of temperature, it had an obvious negative effect on the FIRC of the emulsion gel (*p* < 0.05). To explain further, it would go down as the temperature went from 50 to 100 °C. The mean FIRC occurred after heating the emulsion gel to 50 °C; the emulsion gel achieved a mean FIRC value of 0.12 ± 0.03, significantly greater (*p* < 0.05) than the mean FIRC values of 0.11 ± 0.04, observed at 75 °C, and 0.09 ± 0.04 at 100 °C. This gradual decrease suggests that the components involved in FIRC values in the emulsified system exhibit poor thermal stability.

In summary, this study substantiates the efficacy of an emulsion gel stabilized with CNFs as a protective carrier for fucoxanthin, highlighting its potential for use in functional foods. The structural characteristics of the CNFs, including reduced crystallinity and nanoscale dimensions, were directly responsible for enhancing the interfacial stability and controlling the release of fucoxanthin under various environmental conditions. While the system demonstrated significant antioxidant preservation and dual functionality as both stabilizer and bioactive protector, it also presents limitations related to scalability and industrial compatibility. Future research will prioritize cross-sector collaborations to assess the technological feasibility and production scalability, in addition to conducting pharmacological evaluations such as cytotoxicity and biocompatibility studies to expand applications into pharmaceutical fields.

## 4. Conclusions

In the present investigation, cellulose nanofibers (CNFs) produced from industrial brown seaweed residue via high-pressure homogenization at 105 MPa exhibited a reduced particle size and crystallinity, which directly enhanced their interfacial activity and emulsion gel stabilization capability. The incorporation of fucoxanthin into the CNF-stabilized emulsion gel system not only improved storage stability but also enabled controlled release under diverse environmental conditions including pH, ionic strength, and high temperatures, while effectively preserving antioxidant activity. These findings demonstrate the dual functionality of seaweed-based CNFs as effective stabilizers and bioactive protective agents, offering a sustainable strategy for enhancing the delivery of oxidation-sensitive compounds in functional foods.

This study established stable emulsion gels using cellulose nanofibrils derived from industrial brown seaweed waste to encapsulate oxidation-sensitive fucoxanthin. High-pressure homogenization at 105 MPa over 5–15 cycles yielded the CNFs with a reduced crystallinity down to 52.91% and nanoscale dimensions of 37–91 nm. Single-factor and orthogonal optimization identified 17.5 mg/mL CNFs, 50% coconut oil, 0.500 mg/mL fucoxanthin, and 60 s homogenization as the optimal parameters. Fucoxanthin release exhibited pH, salinity, and temperature dependence, peaking at 9.22% under pH 8.0, 9.52% at 1000 mM NaCl, and 8.25% at 100 °C. The system preserved bioactivity with the DPPH free scavenging rates of 77.64% at pH 8.0 and 75.37% at 1000 mM NaCl, alongside the FIRC of 0.12–0.15%. Seaweed-derived CNFs demonstrated dual functionality by enhancing the emulsion gel stability and protecting bioactive integrity.

This work pioneers a sustainable food-grade delivery platform utilizing seaweed processing waste, offering significant potential for functional foods requiring controlled release and stability during processing and storage. To the best of our knowledge, this is the first study on the fucoxanthin delivery system of emulsion gels stabilized by the CNFs. Future research will therefore prioritize collaborative industrial partnerships to conduct comprehensive feasibility and scalability assessments, ultimately ensuring the seamless adaptation of emulsion technologies for large-scale manufacturing.

## Figures and Tables

**Table 1:** Factors and levels of orthogonal experiment.

Level	Mass Fraction of CNFs (mg/mL)	Homogenization Time(s)	Oil-Phase Volume Fraction (%)
1	12.5	45	47.5
2	15	60	50
3	17.5	75	52.5

**Table 2:** The PDI values and crystallinity index of CNFs *.

Homogenization Cycles	BC	C5	C10	C15
PDI	0.64 ± 0.01 ^a^	0.63 ± 0.03 ^a^	0.62 ± 0.02 ^a^	0.57 ± 0.02 ^b^
CrI/%	60.21 ± 4.32 ^a^	53.22 ± 0.67 ^b^	52.93 ± 2.93 ^b^	52.91 ± 2.13 ^b^

* Different letters (a, b) represent group means that differ significantly (*p* < 0.05).

**Table 3:** The contact angle of the CNFs *.

Homogenization cycles	BC	C5	C10	C15
Interfacial contact angle (°)	44.3 ± 8.5 ^a^	44.5 ± 2.5 ^a^	48.0 ± 1.8 ^a^	45.7 ± 4.4 ^a^

* Different letters (a) represent group means that differ significantly (*p* < 0.05).

**Table 4:** The results of the orthogonal experiment.

Experiment Number	A Oil-Phase Volume Fraction (%)	B Mass Fraction of CNFs (%)	C Homogenization Time(s)	D Creaming Index (%)
1	1	1	1	0.56
2	1	2	2	0.61
3	1	3	3	0.63
4	2	1	3	0.66
5	2	2	1	0.70
6	2	3	2	0.68
7	3	1	2	0.65
8	3	2	3	0.64
9	3	3	1	0.68
K_1_	1.80	1.87	1.94	
K_2_	2.04	1.95	1.94	
K_3_	1.97	1.99	1.93	
k_1_	0.60	0.62	0.65	
k_2_	0.68	0.65	0.65	
k_3_	0.66	0.66	0.64	
R	0.08	0.04	0.01	
Optimal Level	A_2_	B_3_	C_1_	

## Data Availability

The original contributions presented in the study are included in the article, further inquiries can be directed to the corresponding author.
